# Large oncosomes overexpressing integrin alpha-V promote prostate cancer adhesion and invasion via AKT activation

**DOI:** 10.1186/s13046-019-1317-6

**Published:** 2019-07-18

**Authors:** Chiara Ciardiello, Alessandra Leone, Paola Lanuti, Maria S. Roca, Tania Moccia, Valentina R. Minciacchi, Michele Minopoli, Vincenzo Gigantino, Rossella De Cecio, Massimo Rippa, Lucia Petti, Francesca Capone, Carlo Vitagliano, Maria R. Milone, Biagio Pucci, Rita Lombardi, Federica Iannelli, Elena Di Gennaro, Francesca Bruzzese, Marco Marchisio, Maria V. Carriero, Dolores Di Vizio, Alfredo Budillon

**Affiliations:** 1 Experimental Pharmacology Unit, Istituto Nazionale Tumori – IRCCS- Fondazione G. Pascale, Via M. Semmola, 80131 Naples, Italy; 2 Centre on Aging Sciences and Translational Medicine (Ce.S.I.-Me.T.), University “G.d’Annunzio”, Chieti-Pescara, Italy; 3 Department of Medicine and Aging Sciences, University “G. d’Annunzio”, Chieti-Pescara, Italy; 4 Georg-Speyer-Haus Institute for Tumor biology and Experimental Therapy, Frankfurt, Germany; 5 Neoplastic Progression Unit, Istituto Nazionale Tumori – IRCCS- Fondazione G. Pascale, Naples, Italy; 6 Pathology Unit, Istituto Nazionale Tumori – IRCCS- Fondazione G. Pascale, Naples, Italy; 7 Institute of Applied Sciences and Intelligent Systems ‘E. Caianiello’ of CNR, Pozzuoli, Italy; 8 Departments of Surgery, Pathology & Lab Medicine, and Biochemical Science, Cedars-Sinai Medical Center, Los Angeles, CA USA

**Keywords:** Extracellular vesicles, Oncosomes, Prostate cancer, Alpha-V integrin, AKT

## Abstract

**Background:**

Molecular markers for prostate cancer (PCa) are required to improve the early definition of patient outcomes. Atypically large extracellular vesicles (EVs), referred as “Large Oncosomes” (LO), have been identified in highly migratory and invasive PCa cells. We recently developed and characterized the DU145R80 subline, selected from parental DU145 cells as resistant to inhibitors of mevalonate pathway. DU145R80 showed different proteomic profile compared to parental DU145 cells, along with altered cytoskeleton dynamics and a more aggressive phenotype.

**Methods:**

Immunofluorescence staining and western blotting were used to identify blebbing and EVs protein cargo. EVs, purified by gradient ultra-centrifugations, were analyzed by tunable resistive pulse sensing and multi-parametric flow cytometry approach coupled with high-resolution imaging technologies. LO functional effects were tested in vitro by adhesion and invasion assays and in vivo xenograft model in nude mice. Xenograft and patient tumor tissues were analyzed by immunohistochemistry.

**Results:**

We found spontaneous blebbing and increased shedding of LO from DU145R80 compared to DU145 cells. LO from DU145R80, compared to those from DU145, carried increased amounts of key-molecules involved in PCa progression including integrin alpha V (αV-integrin). By incubating DU145 cells with DU145R80-derived LO we demonstrated that αV-integrin on LO surface was functionally involved in the increased adhesion and invasion of recipient cells, via AKT. Indeed either the pre-incubation of LO with an αV-integrin blocking antibody, or a specific AKT inhibition in recipient cells are able to revert the LO-induced functional effects. Moreover, DU145R80-derived LO also increased DU145 tumor engraftment in a mice model. Finally, we identified αV-integrin positive LO-like structures in tumor xenografts as well as in PCa patient tissues. Increased αV-integrin tumor expression correlated with high Gleason score and lymph node status.

**Conclusions:**

Overall, this study is the first to demonstrate the critical role of αV-integrin positive LO in PCa aggressive features, adding new insights in biological function of these large EVs and suggesting their potential use as PCa prognostic markers.

**Electronic supplementary material:**

The online version of this article (10.1186/s13046-019-1317-6) contains supplementary material, which is available to authorized users.

## Background

Prostate Cancer (PCa) is the second most frequent cancer and the fifth leading cause of cancer death in men, mainly due to distant metastasis [1]. The validation of molecular markers able to refine the prognostic prediction of the Gleason score, which is highly imperfect but still the only tool to predict PCa aggressiveness, can improve the early definition of PCa patients clinical outcome, with implications for personalized therapies and follow-up strategies [2]. Both tumor invasion and metastasis are regulated by a complex mechanism finely controlled by the expression and function of adhesion molecules, such as integrins and proteolytic enzymes able to degrade the extracellular matrix (ECM), including metalloproteinases. Integrins belong to a family of 24 heterodimers regulating cells adhesion to the ECM and connect the extracellular space with the cytoskeleton, via their intracellular domains, thus triggering intracellular signaling pathways [3]. Integrins expression is tissue-dependent and it is differentially regulated during either development or functional/physiological changes in normal tissues, while tumor cells are able to modulate integrin expression profiles to enhance their ability to migrate, invade, metastasize, and survive in hostile environments [3]. Intriguingly, integrins relevance in cancer progression has been recently recognized when expressed on extracellular vesicles (EVs) surface [4–6]. EVs are considered as one of the most effective vehicles of information among cells and recent findings demonstrated that they play an important role in cancer development and progression, by transferring information between cancer cells as well as between cancer cells and tumor microenvironment, at both paracrine and systemic level [7, 8]. Although highly heterogeneous, two major classes of EVs have been described: the exosomes, small size vesicles (30–150 nm) generated through the classical endosome-multivesicular body pathway, and shed microvesicles, formed through the direct budding of the plasma membrane. Among various populations of this latter class of EVs, atypically large EVs (1–10 μm in diamter) referred as “Large Oncosomes” (LO) have been identified in highly migratory and invasive PCa cells [9, 10]. LO-like structures have been found in tumor tissues and plasma of metastatic PCa patients and have not been detected in benign tissues [11, 12]. LO can contribute to tumor progression because are able to degrade directly ECM in vitro and few reports suggest they can also export specific oncogenic cargo to other tumor or stroma cells, reprogramming their phenotype, thus establishing a tumor growth-supporting environment [11–13]. Our group has recently developed an isogenic model, consisting of the parental DU145 PCa cell line and their derived aggressive-resistant subline DU145R80, generated through a stepwise selection with increasing concentrations of zoledronic acid (ZOL), a nitrogen containing bisphosphonates employed in clinical practice to reduce skeletal complications related with bone metastasis of several cancer, including PCa, but also able to exert a direct antitumor activity [14, 15]. We and others have shown that ZOL effect is mediated through the inhibition of the mevalonate pathway [15], indeed DU145R80 are also cross-resistant to statins.^1^ DU145R80 cells show a highly aggressive phenotype consisting of increased invasive capability, epithelial to mesenchymal transition (EMT), enrichment in cancer stem cells (CSC) markers and altered cytoskeleton organization as well as a different protein profile compared to parental DU145 cells [14, 16, 17]. We previously demonstrated that integrin alpha V (αV-integrin), which is linked to cancer progression in PCa and other tumors, was upregulated in DU145R80 cells and functionally involved in their increased invasive phenotype compared to parental DU145 cells [16, 17].

In the present study, by using the DU145/DU145R80 system, we defined a paracrine effect exerted by αV-integrin-overexpressing LO to promote tumor aggressiveness of PCa cells via AKT activation.

## Materials and methods

### Cell culture

The PCa cell line DU145 was purchased from American Type Culture Collection (Rockville, MD, USA). DU145R80 cells were obtained by treating DU145 with increasing concentrations of ZOL as previously described [14]. Both DU145 and DU145R80 cells were cultured in RPMI 1640 (Lonza) - containing 10% of heat-inactivated fetal bovine serum (FBS; Lonza), 10000 U/ml penicillin and 10 mg/ml streptomycin (Lonza), 20 mM Hepes (pH 7.4) and 4 mM L-glutamine; in a humidified atmosphere composed of 95% air and 5% CO_2_ at 37 °C. Cell lines were regularly inspected for mycoplasma.

### Membrane labeling

Cells (3,5 × 10^4^) were allowed to adhere on glass coverslips at 37 °C in humidified air with 5% CO_2_. After 4 h of serum free media, cells were incubated with FITC-conjugated cholera toxin B subunit for 5 min on ice, following the manufacturer instructions (CTxB, Sigma, C1655, 1:1000) [13]. Cells were then fixed with 4% paraformaldehyde (PFA) for 10 min at RT. After several washes in PBS, slides were mounted using a mounting solution with DAPI, and were visualized by fluorescent microscopy.

### Isolation of large EVs and LO purification

Large EVs were purified by differential low speed centrifugation (10,000 g for 30 min) of conditioned media from cells cultured for 24 h in serum free medium. LO purification was performed in discontinuous gradient of iodixanol (OptiPrep™ by Fresenius Kabi Norge AS for Axis-Shield PoC AS, Oslo, Norway) [12, 13, 18]. In detail, the 10,000 g pellets were mixed in the bottom layer (60% w/v iodixanol, indicated in figures as 1.32 g/mL density fraction) and the gradient solutions were layered carefully on the top; then centrifugation was performed at 100,000 g for 3:50 h at 4 °C and eight individual fractions were collected and washed with PBS. After centrifugation at 100,000 g for 1 h at 4 °C, pellets from each fraction were suspended in either PBS for functional assays or NP40 lysis buffer (Life Technologies, Frederick, MD 21704, USA) for protein studies.

### Tunable resistive pulse sensing measurements

LO preparations (see above) from ~ 200 × 10^6^ of each cell line, suspended in 0.22 μm filtered PBS, were submitted to tunable resistive pulse sensing (TRPS) analysis using a qNano instrument (IZON Science, Ltd., Christchurch, New Zealand) as described for LO previously [12].

### LO optical images

LO images were acquired by Olympus BX51 optical microscope, in upright configuration. In details, LO suspended in 0.22 μm filtered PBS were imaged in dark field in transmission using an Olympus U-DCD condenser and an objective 50× (N.A. = 0.75). Bright field images were achieved using lateral illumination at 50× (N.A. = 0.75) and a 100× (N.A. = 0.9), respectively.

### Flow cytometry analysis

Detection of EVs > 1 μm: flow cytometry quantitative analysis was first performed on large EVs isolated from conditioned medium of 24 h serum free cultured cells (~ 30 × 10^6^) and suspended in 0.22 μm filtered PBS; at least 3000 events were acquired and analyzed using the BD LSRII flow cytometer and FlowJo software; bead standards of 1, 2, 4, 6, and 10 μm were used [11].

Detection of fluorescent- labelled, iodixanol purified LO: flow cytometry on fluorescent- labelled LO was performed using LO preparations from ~ 60 × 10^6^ cells, suspended in 0.22 μm filtered PBS, and incubated with a fluorescent phycoerythrin (PE)-conjugated anti-αV-integrin antibody (1:10 anti-PE CD51 by Biolegend, San Diego, CA), 1 μl of 0.2 mM lipophilic cationic dye (patent pending, code: 102018000003981), plus Phalloidin-FITC (1:100 by Merck KGaA, Darmstadt, Germany), incubated for 30 min at 37 °C; after fixation and permeabilization 10 min each at RT, LO were incubated with anti-PerCP CK18 antibody (1:25 by Abcam, Cambridge, England) for 30 min at 37 °C; 1 × 10^6^ events/sample were acquired by flow cytometry (FACSVerse, BD™), by setting a low threshold (200) on the channel in which the lipophilic cationic dye emits (APC-H channel); no threshold on morphological parameters was applied and amplifier settings for forward scatter (FSC) and side scatter (SSC) as well as for any fluorescence channel were set in logarithmic mode. LO morphology was confirmed by running both Megamix Plus (FSC and SSC) and Spherotech beads at the same photomultiplier (PMT) voltages used for LO detection. In order to evaluate non-specific fluorescence, Fluorescence Minus One (FMO) controls were used as the best control in a multicolor staining combination instead of isotype controls, as already reported by other groups [19, 20]. Compensation was assessed using CompBeads (BD™) and single stained fluorescent samples. Data were analyzed using FACSuite v 1.0.5 (BD™) software.

For the gating strategy, we used both Megamix Plus (Biocytex, Marseille, France) and Spherotech beads (Spherotech Inc., Lake Forest, IL, USA). The area containing the 0.1–1 μm size events (“microvesicles area”) as well as the 1–10 μm size events (“LO area”) were identified on a dot-plot FSC-height (FSC-H) versus SSC-height (SSC-H). For each analyzed sample (acquired with the same settings used for beads), intact events (Phalloidin negative), falling in the gate of “LO area” and positive for the lipophilic cationic dye (LCD, which identifies the whole extracellular vesicle compartment) have been analyzed for CK18 and αV-integrin.

Imaging cytometry of fluorescent-labeled iodixanol purified LO: acquisition was performed using ImageStreamX Imaging Flow Cytometer (Amnis Corporation, Seattle, USA) equipped with INSPIRE software. All samples (LO145 and LOR80) were incubated for 1 h at 37 °C with the phycoerythrin (PE)-conjugated anti-αV-integrin antibody and 5000 events/sample were acquired by a 40× magnification objective. Data analysis was performed using the IDEAS software (Amnis Corporation). PE fluorescence was excited with a 100 mW of 488 nm argon laser and collected on channel three (560–595 nm). Intensity adjusted bright field images were collected on channel four.

### Immunofluorescence of specific proteins

Cells (~ 3 × 10^4^/sample) were seeded on glass coverslips and cultured for 24 h in growth medium. Then, slides were washed with PBS, fixed with 2.5% formaldehyde in PBS for 10 min at 4 °C and then incubated with 2 μg/mL anti-R4 anti-uPAR monoclonal antibody recognizing uPAR D3 domain (1 μg/mL) [18], 2 μg/mL rabbit anti-αV polyclonal antibody from Abcam (EPR16800) for 2 h at RT, permeabilized with 0.1% Triton X-100 in PBS for 10 min at 4 °C and then incubated for additional 2 h at RT with 2 μg/mL guinea pig anti-cytokeratin 18 polyclonal antibody (Progen) (Fig. 3a). Anti-αV polyclonal antibody from Abcam, permeabilization step and guinea pig anti-cytokeratin 18 (CK18) were employed for Fig. 3b. Then, after three washes with PBS, 1:800 Alexa Fluor 488-conjugated F(ab’)2 fragment of rabbit anti-rabbit IgG, or Alexa Fluor 594 goat anti-guinea pig IgG antibodies, both purchased from Molecular Probes were applied to slides at RT for 45 min. After nuclear staining with 4–6-diamidino-2-phenylindole dye (DAPI), coverslips were mounted using 20% (w/v) mowiol and analyzed by a fluorescence inverted microscope connected to a video-camera (Carl Zeiss) or by the LMS510 Zeiss confocal microscope (Carl Zeiss).

### Immunoblotting

Proteins from both cells lysates and LO lysates were analyzed by SDS-PAGE and Western Blot (WB) performed with antibodies as indicated in the figures (detailed information on antibodies are presented in Additional file 1). Enhanced chemiluminescence (ECL) immunodetection reagents were from GE Healthcare. The chemiluminescent signal was detected with Image Quant LAS 500, and the intensity was measured by ImageQuantTL image software (GE Healthcare). Image J was used to quantify protein bands from western blot images. The quantification reflects the relative amounts as a ratio of each protein band relative to loading control (γ- tubulin).

### Adhesion assay

Cell adhesion assays were performed using 24-well tissue culture plates uncoated/coated with vitronectin (VN) (Corning, NY 14831 USA). In details, plates were incubated ON with VN (2,5 μg/mL in PBS) at 4 °C. After gentle washing with PBS, 1 h BSA1% incubation at RT and again washing with PBS, recipient cells treated with either PBS or LO (20 μg/mL) were plated in each well (6 × 10^4^ viable cells/well) and incubated at 37 °C, 5% CO2 in serum-free medium. When indicated, the adhesion assay was preceded by a pre-treatment: LO pre-treated for 45 min with 1:500 diluted blocking anti-αV monoclonal antibody, clone 272-17E6 (Merck Millipore, Burlington, Massachusetts, USA); or recipient cells with GDC-0068 AKT inhibitor (Ipatasertib by Selleckchem, Munich, Germany). Dosage and time of treatment as indicated in figures. As negative controls for either anti-αV-pre-treated LO, or GDC-0068 pre-treated recipient cells, cells were treated with anti-αV monoclonal blocking antibody or AKT inhibitor alone, respectively. At the indicated times, the adherent cells were counted and arbitrary values of 100% was given to the basal adhesion of cells treated with only PBS and values were reported relative to that.

### Invasion assay

Invasion assays were performed in Boyden chambers, using 8 μm pore size PVPF filters (Nucleopore, GE Healthcare, Chicago, Illinois, USA) coated with 50 μg/filter matrigel (BD Biosciences, Two Oak Park, Bedford, MA) as previously described [17]. Briefly, 1.5 × 10^4^ viable cells (pre-treated with GDC-0068 AKT inhibitor, only when specifically indicated in figure) were exposed to LO derived from either DU145 (LO145) or DU145R80 (LOR80) cells and right after, treated cells were seeded in the upper chambers in serum-free medium. LO were untreated/pre-treated with 1:500 diluted blocking anti-αV monoclonal antibody or anti-Pan Akt monoclonal antibody (R&D systems, Minneapolis, Minnesota, USA), the last used as antibody negative control. The lower chambers were filled with RPMI plus/minus 10% serum. Cells were allowed to invade matrigel for 16 h at 37 °C, 5% CO2. At the end of the assay, cells on the lower filter surface were fixed with ethanol, stained with haematoxylin and 10 random fields/filter were counted at 200× magnification. The arbitrary value of 100% was given to basal invasion of recipient PBS-treated cells, all values were reported relative to that.

### In vivo experiments

Female CD1 athymic nude mice (Charles River) were acclimatized in the Animal Care Facility of “Fondazione G. Pascale-IRCCS-CROM Laboratories”, in accordance with “Directive 2010/63/EU on the protection of animals used for scientific purpose” and made effective in Italy by the legislative DLGS 26/2014. The study was approved by the Italian Ministry of Health. DU145R80 (5 × 10^6^) and DU145 (5 × 10^6^) cells both in serum free RPMI were injected subcutaneously in the flank of mice. DU145 were recombined without or with alternatively LO145 or LOR80. DU145 cells pretreatment with LO was performed at a concentration of 80 μg/mL. Tumor size was measured twice a week and calculated as: ½ × width^2^ × length. Animals were monitored for abnormal tissue growth and euthanized if excessive health deterioration was observed.

### Patient specimens and tissue microarray (TMA)

A series of 103 patients who underwent radical prostatectomy at Istituto Nazionale Tumori – IRCCS – Fondazione G. Pascale, Napoli (Italy) between 2005 and 2014 were retrieved (Patient’s characteristics in Additional file 8: Table S1). The approval of Institute Independent Ethical Committee was given (BioPro study). Sections of 4 μm thickness from each block were obtained and were stained with hematoxylin-eosin. All cases were reviewed by pathologists according with WHO classification.

TMA were built using 103 Prostate Cancer tissue samples mentioned above. Two tissue cylinders for every single patient with a diameter of 1 mm were punched from morphologically representative tissue areas of each ‘donor’ tissue block and brought into one recipient paraffin block (3 × 2.5 cm) using a semi-automated tissue arrayer (CK 3500 Tissue Microarrayer, Galileo TMA, Integrated System Engineering).

### Immunohistochemistry analysis

Tumor tissues were stained with either hematoxylin and eosin or immunostained with αV-integrin (EPR16800 Abcam Rabbit monoclonal IgG 1:500) or CK18 (Monoclonal Mouse M7010 Clone D10 1:500 Dako). In details, paraffin slides were de-paraffinized in xylene and rehydrated through graded alcohols. Antigen retrieval was performed with slides heated in 0.01 M citrate buffer (pH 6.0) in a bath for 20 min at 97 °C. After antigen retrieval, the slides were allowed to cool. The slides were rinsed with TBS and the endogenous peroxidase was inactivated with 3% hydrogen peroxide. After protein block (BSA 5% in PBS), the slides were incubated with the above mentioned primary antibodies. The sections were rinsed in TBS and incubated for 20 min with Novocastra Biotinylated Secondary Antibody (RE7103), that recognized mouse and rabbit immunoglobulins. Then the sections were rinsed in TBS and incubated for 20 min with Novocastra Streptavidin-HRP (RE7104) and then peroxidase reactivity was visualized using a 3,3′-diaminobenzidine (DAB) (Sigma Aldrich). Results were interpreted in accordance with the pathologists, using a light microscope.

### Statistical analysis

Results of in vitro cell assays are expressed as the mean for at least three independent experiments, which were conducted in triplicate (±SD), unless differently specified in figure legend. Representative results from a single experiment of qRT-PCR, WB and immunohistochemistry (IHC) are showed; additional experiments yielded similar results. Statistical analysis by t-Student test was performed: ***p ≤ 0,001; **p ≤ 0,005; *p ≤ 0,05 (unless where it is differentially mentioned in figure legend). Statistical significance for tumor incidence in mice was also calculated by t-Student, while mice survival by Gehan-Breslow-Wilcoxon test. For IHC evaluation on human tissue samples, differences in the expression of αV-integrin according to clinic and pathological features were evaluated using Kruskal–Wallis tests.

Additional information are available in Additional file 1: Supplementary Methods.

## Results

### Increased spontaneous bleb formation and large EVs shedding by DU145R80 cells

**Fig. 1:**
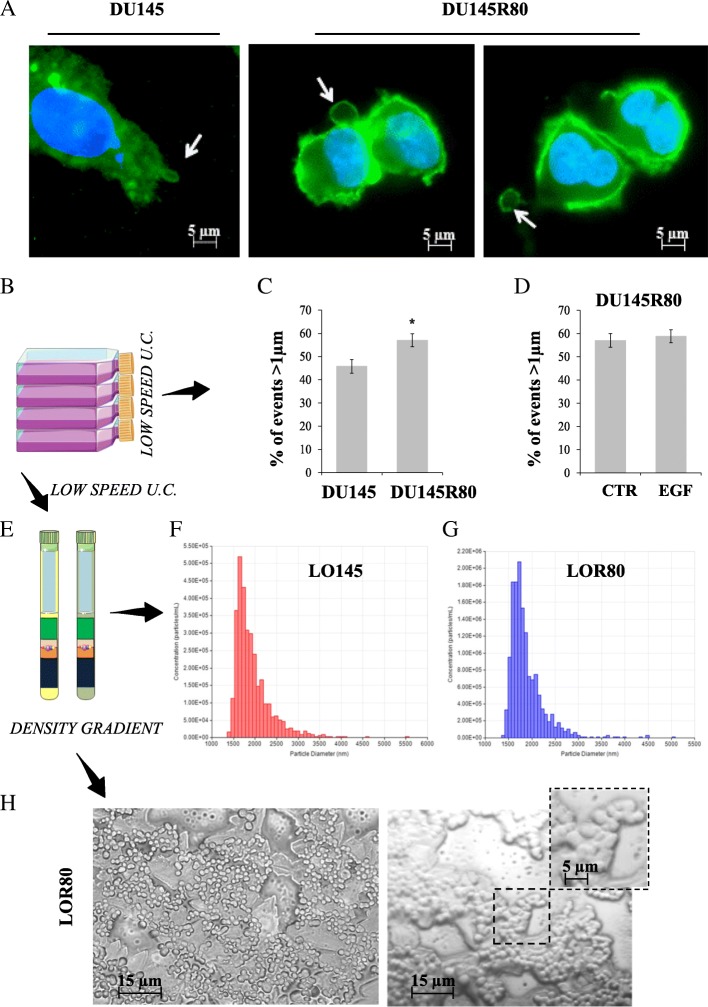
Spontaneous blebs formation and increased EVs shedding in DU145R80 compared to DU145 cells. **a** Fluorescent microscopy of DU145 and DU145R80 cells obtained after by FITC-CTxB membrane labelling and imaged at 40× magnification. **b-c** Flow cytometric detection of large EVs spontaneously shed by either DU145 or DU145R80 and collected by low speed centrifugation from conditioned serum free media. **d** Large EVs shedding by untreated or EGF-stimulated DU145R80 cells. **e** Scheme of iodixanol purified fractions of large EVs from both cell lines. **f**, **g** Tunable resistive pulse sensing analysis (by qNano Instrument) of LO from DU145 (red) and DU145R80 (blue) using NP2000 membrane pores. **h** LO from DU145R80: representative images by optical microscopy (50× and 100× without or with zoom magnifications)

DU145R80 cells cultured in serum free media produce spontaneously large EVs (blebs, 1–10 μm in diameter), resembling those previously referred as LO [10] (Fig. 1a). DU145 parental cells demonstrated less spontaneous blebbing compared to DU145R80 in agreement with a previous report demonstrating in this cell line blebbing only upon EGF treatment [10] (Fig. 1a). We also demonstrated a spontaneous, significant increase in large EVs shedding from DU145R80 compared to DU145 cells, by flow cytometry analysis considering only size and number of events (Fig. 1b-c). EV shedding by DU145R80 was not due to an increased rate of apoptosis (Additional file 2: Figure S1), suggesting that these EVs originate from non-apoptotic membrane blebs, as previously described for LO [9]. However, differently for what has been reported for LO in the DU145 model [9, 10], the number of EVs > 1 μm in DU145R80 was not increased by treatment with 50 ng/mL epidermal growth factor (EGF) (Fig. 1d). Next, large EVs from both DU145 (LO145) and DU145R80 (LOR80) cells were purified by discontinuous iodixanol (OptiPrep™) density gradient and the LO-enriched fractions (1.15 g/ml) [13] were analyzed by tunable resistive pulse sensing (TRPS) (Fig. 1e-g). We identified a population of EVs with heterogeneous diameters ranging from 1.5 to 5–6 μm confirming the large size of EVs identified by flow cytometry. Moreover, TRPS data showed that the number of large EVs in LOR80 was enriched in comparison to LO145. Finally by analyzing the same fractions by optical microscopy imaging, we revealed the presence of EVs of 2–4 μm diameter (Fig. 1h), again confirming TRPS data.

### Selective packaging of protein cargo in LO shed by DU145R80

**Fig. 2:**
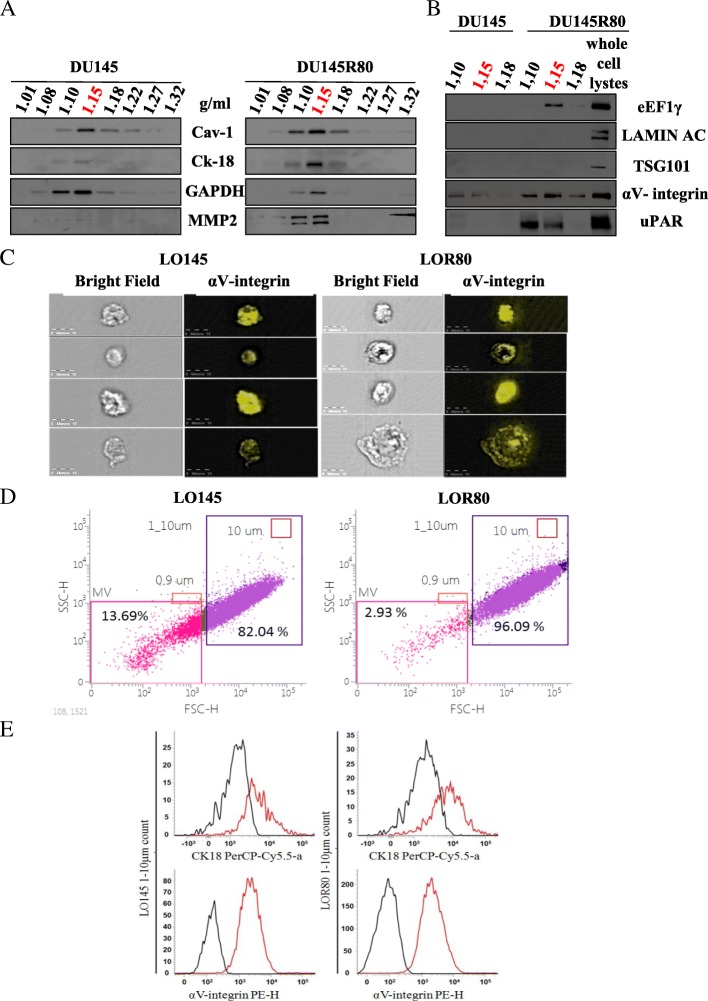
DU145 and DU145R80-derived LO protein cargo. **a-b** Western blot analysis for the indicated proteins on iodixanol purified fractions of large EVs from both cell lines. Fractions enriched in LO are indicated in red. Whole cell lysates from DU145R80 used as positive control. **c** αV-integrin expression on LO145 (left panels) and LOR80 (right panels), purified by iodixanol gradient (1.15 g/mL fractions), measured by Amnis® Imaging Flow Cytometer upon labeling with a specific PE-conjugated antibody. Representative images were acquired at 40× magnification (bar size 10 μm). **d** Flow cytometric analysis of both LO145 and LOR80 purified by iodixanol gradient (1.15 g/mL fractions), identified as intact vesicles in a range size between 1 and 10 μm and positive for αV-integrin. **e** Flow cytometric analysis of LO145 and LOR80 purified by iodixanol gradient (1.15 g/mL fractions) and positive (red) for CK18 (upper panel) and αV-integrin (lower panel) as compared to negative controls (black)

We demonstrated by western blot (WB) analysis that the iodixanol EVs fractions floating at a buoyant density of 1.10–1.15 g/ml, from both DU145 and DU145R80 cells, were enriched in proteins such as caveolin 1 (Cav-1), CK18 and GAPDH (Fig. 2a), which have been previously demonstrated to be abundant in LO [13], and were negative for TSG101, which is typically enriched in exosomes (Fig. 2b). We also looked at expression of proteins identified as differentially expressed in DU145R80 versus DU145 cells in our previous mass spectrometry study [16, 17]. We observed that matrix metalloproteinase 2 (MMP2) (Fig. 2a), urokinase-type plasminogen activator receptor (uPAR), αV-integrin and eukaryotic elongation factor 1 gamma (eEF1γ) were expressed at significant higher levels in LO fractions from DU145R80 compared to DU145 cells (Fig. 2b). However, lamin AC, which is upregulated in DU145R80 cells and involved in malignant behavior of PCa [17], was not found in LO fractions, despite its presence has been observed in other populations of cancer-derived EVs [19], confirming a specific proteins packaging of LO by cancer cells (Fig. 2b). Since αV-integrin emerged as a key player in the acquisition of DU145R80 aggressive phenotype [17], we next analyzed αV-integrin expression on LO145 and LOR80, labeled with a specific PE-conjugated antibody, by the Amnis® imaging flow cytometer. This analysis confirmed the large size of this population of EVs and showed a “patchy” expression pattern of αV-integrin on the surface of LO from both cell lines, suggesting a polarized organization that well correlates with integrins clustering and activation [3] (Fig. 2c).

LO from both cell lines were then analyzed by a recently developed flow cytometry-based strategy able to identify undamaged and vital EVs (patent pending, see methods section, *P. Lanuti* et al. *Manuscript submitted*). Intact and vital αV-integrin positive-LO were confirmed to be of heterogeneous size, ranging between 1 and 10 μm and those derived from DU145R80 (LOR80) resulted significantly enriched in LO (96%) compared to those from DU145 cells (LO145) (82%) (Fig. 2d), confirming data presented in Fig. 1. Moreover, both LOR80 and LO145 resulted clearly positive for both αV-integrin and the LO marker CK18 (Fig. 2e). The mean fluorescent intensity (MFI) of both αV-integrin and CK18 was significantly higher in LOR80 compared to LO145 (*data not shown*).

**Fig. 3:**
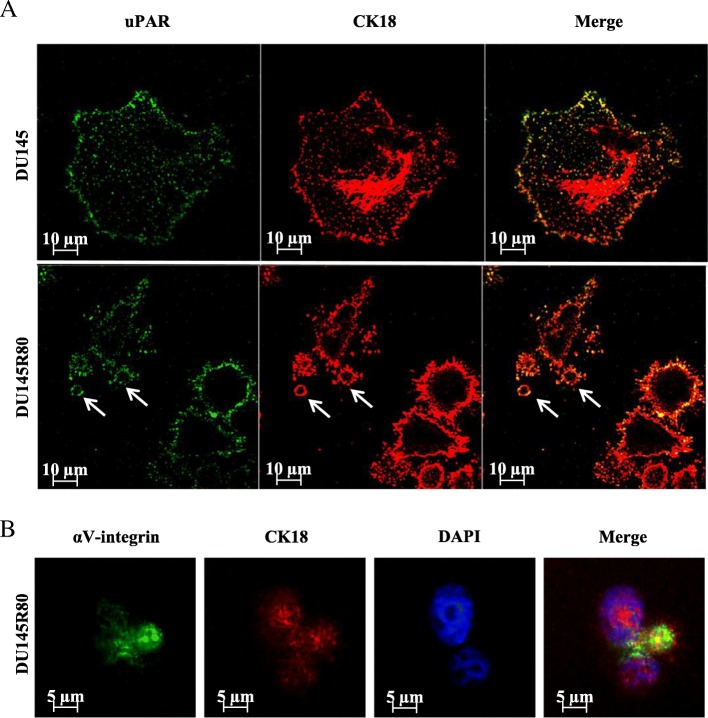
Immunofluorescence analysis of DU145 and DU145R80 cells. **a** Confocal microscopy analysis of uPAR and CK18 in DU145 and DU145R80 cells. Original magnification 63×. LO-like vesicles (about 5–6 μm) shed from DU145R80 cells are indicated with arrows (lower panel). **b** Fluorescence microscopy analysis of αV-integrin- and CK18-positive 5–6 μm LO-like vesicles arising from DU145R80 cells plasma membraner

LO-like vesicles (about 5–6 μm), shed from DU145R80 cells and positive for LO marker CK18 and for uPAR were highlighted also by confocal microscopy (Fig. 3a, arrows). Moreover, a clear co-localization of CK18 and of uPAR, both of them previously identified in LO fraction by WB, was recognized at the cell periphery and on blebs/LO surface in DU145R80 cells unlike DU145 cells (Fig. 3a). Furthermore, we also demonstrated that CK18 co-localize with αV-integrin on 5–6 μm blebs/LO arising from the plasma membrane of DU145R80 cells (Fig. 3b). Interestingly, a colorimetric assay confirmed a significant overexpression of αV compared with other integrins, on the surface of DU145R80 vs DU145 cells (Additional file 3: Figure S2), confirming our previous data [17].

### αV-integrin on the surface of DU145R80-derived LO was functionally involved in the increased adhesion and invasion of recipient cells, via AKT-dependent activation

**Fig. 4:**
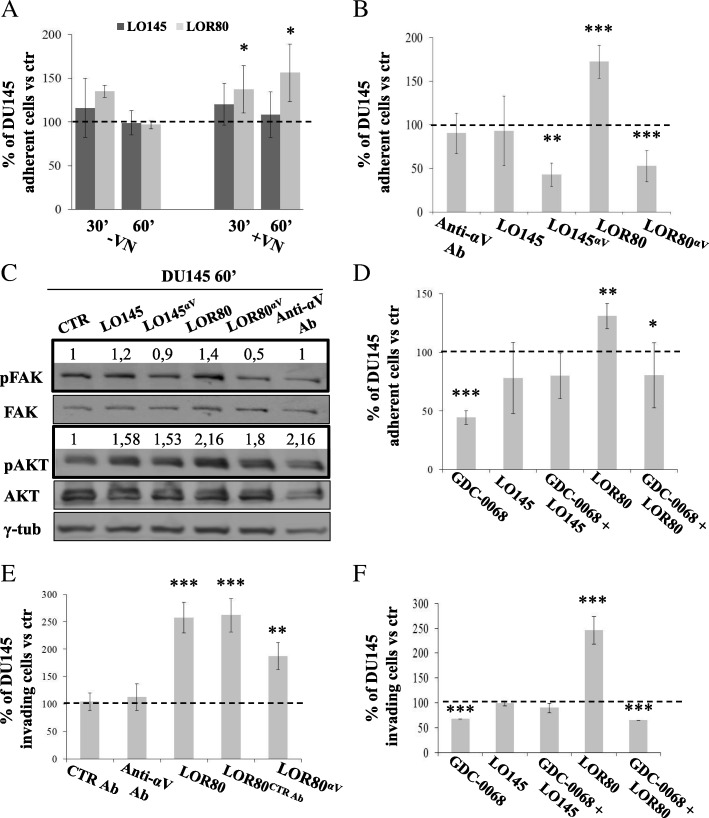
DU145 cells adhesion, invasion and intracellular activated pathways upon exposure to LO145 or LOR80. **a** DU145 adhesion, expressed as % of adherent cells, upon exposure to LO145 or LOR80 vs vehicle (considered as 100% control) and evaluated both in absence or presence of vitronectin (VN) for either 30 or 60 min. Stat. significances are expressed on vehicle. **b** DU145 adhesion measured as above, on VN after 60′ of exposure to LO145 or LOR80, untreated or pre-treated for 45′ RT with anti-αV-integrin monoclonal blocking antibody (anti-αV Ab) and indicated as LO145^αv^ and LOR80^αv^, respectively; as negative control cells were treated by the anti-αV Ab alone. (LO145^αv^/ LO145)**; (LOR80^αv^/ LOR80)***; (LOR80/ vehicle) ***. **c** Western blotting analysis of pFAK, FAK, pAKT, AKT and γ-tubulin on cell lysates from DU145 untreated (CTR), or treated with LO145 or LOR80, alone or pre-exposed to anti-αV Ab (see above). pFAK and pAKT quantification is also reported. **d** Adhesion of DU145 measured as above, untreated (considered as 100%) or pretreated with the AKT inhibitor GDC-0068 for 16 h, were exposed to vehicle, LO145 or LOR80. (GDC-0068/ vehicle)***; (LOR80/ vehicle) **; (GDC-0068+ LOR80/ LOR80)* (**e**) DU145 invasion, expressed as % of invading cells, upon 16 h exposure to LOR80 treated with anti-αV Ab (LOR80^av^) or control Ab (LOR80^CTR Ab^) vs vehicle (considered as 100% control); as negative control cells were treated by CTR Ab alone (anti-pAKT antibody ineffective on non-permeabilized intact cells); (LOR80/ vehicle)***; (LOR80^CTR Ab^*/*vehicle)***; (LOR80^av^*/*LOR80)** (**f**) Invasion of DU145 measured as above, untreated (considered as 100%), or pretreated with the AKT inhibitor GDC-0068 for 6 h and exposed to vehicle, LO145 or LOR80;(GDC-0068/ vehicle)***; (LOR80/ vehicle)***; (GDC-0068 + LOR80/ LOR80)***. Results are representative of a single experiment and several experiments yielded similar results (**b**), or are the average of at least two experiments (**a**; **d**; **e**; **f**)

In order to study the functional role of LO and of their molecular determinants described above, we first explored the adhesion capability of DU145 cells pre-exposed to LO145 or LOR80, respectively. We found that LOR80 triggered a statistically significant increase of DU145 cell adhesion onto vitronectin (VN) within 30 min, that further increased after 60 min, whereas LO145 promoted only a slight non-statistically significant effect after either 30 or 60 min (Fig. 4a). The finding that LOR80 promote DU145 cell adhesion onto VN at a significantly larger extent than LO145 encouraged us to explore the role of αV-integrin, a component of the multiple VN interactor complexes [20], in the LO-induced DU145 cell adhesiveness after 60 min. As shown in Fig. 4b, pre-treating LO with αV-integrin blocking antibody resulted in a dramatic reduction of both LO145 and LOR80-dependent DU145 cell adhesion onto VN. Since pre-incubation of DU145 cells alone with the anti-αV antibody did not fully abrogated their adhesive capability (Fig. 4b), it is conceivable to hypothesize that LO might play an active role in intercellular transfer of cell adhesion properties and that this process is mediated by αV-integrin expressed on the LO surface.

In line with these results, we observed increased phosphorylation of both AKT and focal adhesion kinase (FAK), which are key components of the signal transduction pathway involved in the integrin mediated-adhesion process [3, 21], in DU145 cells exposed to LOR80 compared to those exposed to LO145. The activation of the FAK-AKT signaling pathway was evident within 15 min and up to 1 h from LO treatment (Fig. 4c, Additional file 4: Figure S3A) and was completely reverted by pre-treating LO with the αV-integrin blocking antibody (Fig. 4c). Notably, MAPK was not activated in DU145 exposed to LO (Additional file 4: Figure S3A). Moreover, we also demonstrated that blocking AKT activation in DU145 recipient cells by pre-treatment with the specific AKT-inhibitor GDC-0068 [22] (Additional file 4: Figure S3B), reverted LOR80-induced cell adhesion onto VN (Fig. 4d). Similar results were obtained by using LNCaP as recipient cells, confirming that LOR80 were able to enhance cell adhesion onto VN and that this effect was AKT dependent (Additional file 4: Figure S3C).

Next we explored the invasion capability of DU145 cells pre-exposed to either LOR80 or LO145. By using Boyden chambers and filters coated with matrigel we observed a significant pro-invasive ability induced by LOR80 on DU145 cells (Fig. 4e) while LO145 only elicited a minor effect (Fig. 4f and Additional file 5: Figure S4A). Again, an induction of invasion was also observed by using LNCaP as recipient cells, upon treatment with either LO145 or LOR80 (Additional file 5: Figure S4B). Conversely, no significant pro-invasive effect was observed by pre-exposing normal epithelial prostate (EPN) cell line with LO (Additional file 5: Figure S4B), suggesting that the observed LO-induced functional effects are confined to tumor cells.

Interestingly, 6 h pre-exposure of DU145 recipient cells with LOR80 resulted also in a significant increase of matrix metalloproteinase 9 (MMP9) (Additional file 6: Figure S5A) and in a weak increase of MMP2 (data not shown) mRNA expression, compared with no effect induced by LO145. Accordingly, gel zymography assay showed that DU145, treated with LOR80, released a greater amount of bioactive MMP2 compared to DU145 treated with LO145 (Additional file 6: Figure S5B). Basal MMP2 activity of DU145R80 cells was also higher compared with basal and 0.5% EtOh-treated MMP2 activity [23] of DU145, confirming our previous findings demonstrating overexpression of MMP2 by DU145R80 vs DU145 cells [14] (Additional file 6: Figure S5B).

The LOR80 pro-invasive effect was significantly but not completely reverted by the pre-incubation of LO with an αV-integrin blocking antibody (Fig. 4e). We also observed that pre-incubation of either LOR80 or LO145 with αV-blocking antibody produced a significant decrease in the amount of active MMPs released by DU145 recipient cells compared with those incubated with untreated LO (Additional file 6: Figure S5C). Moreover, when DU145 recipient cells where pretreated for 6 h with the AKT-inhibitor GDC-0068, LOR80-induced invasion was completely reverted (Fig. 4f).

Finally, in line with the previous observations reporting that LO elicit a direct bioactive role and can degrade extracellular matrices in vitro [11] we also confirmed that both LOR80 and LO145 have a gelatinase activity that can be prevented by a pre-incubation with proteases inhibitors (P.I.) (Additional file 6: Figure S5D), as already observed for LO from different sources [11]. Notably, the size range (2 to 4 μm) and modal distribution of the degradation spots observed by the fluorescent gelatin matrix assay were comparable to those previously described for LO activity (Additional file 6: Figure S5E), but also to LOR80 and LO145 size data reported in Fig. 1. Moreover, areas of proteolytic clearance in the fluorescent gelatin, expressed as percentage of degradation spots, were almost doubled when gelatin was exposed to LO from DU145R80 compared to those from DU145 cells (Additional file 6: Figure S5F).

### DU145R80-derived LO promote tumorigenesis of xenograft DU145 cells in nude mice

**Fig. 5:**
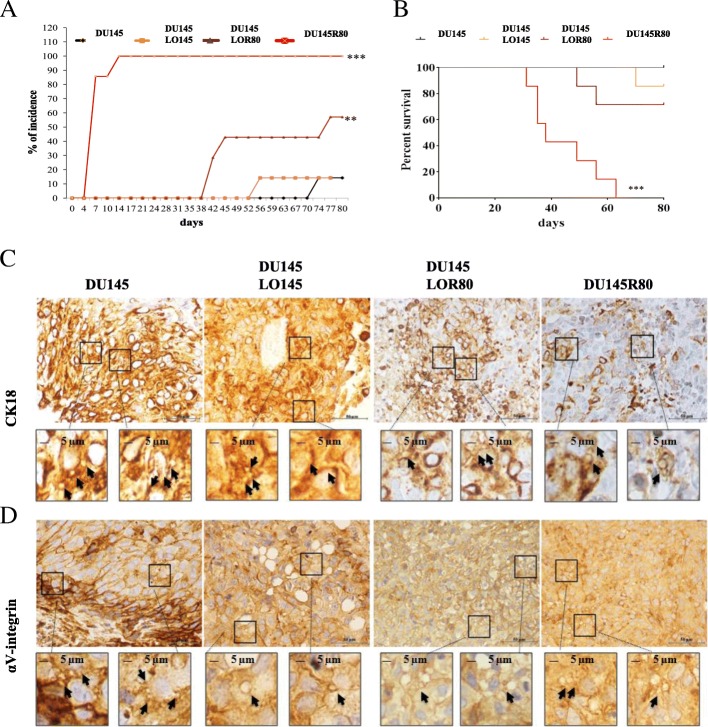
DU145R80-derived LO promote DU145 tumor engraftment in xenograft nude mice and have been found in tumor tissues from mice. **a** Tumor incidence in mice injected with DU145 cells (1/7), DU145R80 cells (7/7), DU145 cells treated with 80 μg/μL of either LO145 (1/7) or LOR80 (4/7). T-Student test: ***p < 0.0001 (DU145R80 vs all groups), **p = 0.0001 (LOR80 vs DU145). **b** Survival of the mice xenografted with the different cell lines by Kaplan-Meyer analysis. ***p < 0.0001 (**c**) CK18 and (**d**) αV-integrin immunohystochemistry staining of xenograft tumours. Arrows indicate LO-like vesicles of 1–5 μm diameter

We next tested the role of LOR80 treatment on the growth of DU145 xenograft tumors injected subcutaneously in nude mice. Notably, 4/7 (57%) mice injected with LOR80-treated DU145, developed tumors with a latency of 42 days, whereas only 1/7 (14,2%) mice developed tumors when injected with DU145 alone (after 74 days), or LO145-treated DU145 (after 59 days) (Fig. 5A). All the animals injected with DU145R80 developed rapidly growing tumors (100% incidence) within 14 days, confirming also in vivo the aggressive behavior previously reported in vitro for this cell line [14, 16, 17]. Survival curves were directly correlated with tumor growth behavior (Fig. 5b). Although DU145R80 xenograft tumors become palpable in few days, and LO-treated DU145 xenograft only after few weeks, the hematoxylin and eosin (H&E) stain features and growth kinetics of the tumors appear similar in all groups (Additional file 7: Figure S6A-B). More importantly, by immunohistochemistry (IHC) staining of tumor tissues from xenograft models with either CK18 (Fig. 5c) or αV-integrin (Fig. 5d) we were able to detect LO-like structures ranging 2–5 μm in size (see arrows in 60× panels, Fig. 5c and d).

In summary, our data on xenograft tumor model confirmed the aggressive feature of DU145R80 cells, demonstrated an in vivo tumor-promoting activity of LOR80, and identify for the first time αV-integrin-positive LO like structures in xenograft tumor tissues.

### αV-integrin expression and LO-like vesicles in human PCa tissues

**Fig. 6:**
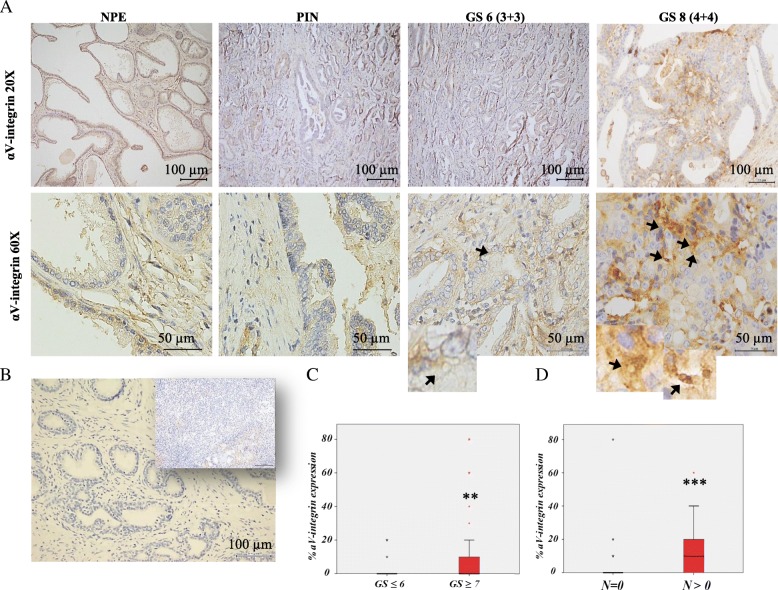
αV-integrin detection in human PCa tissues reveals LO like vesicles and correlates with prognosis. αV- integrin staining of human PCa tissues, performed by immunohystochemistry. **a** Benign tissues (Normal Prostate Epithelia, NPE), prostatic intraepithelial neoplasia (PIN); tumor tissues (Gleason Score = 6; Gleason Score = 8, GS6 and GS8, respectively). All images are 20× magnified in the upper panels; 60× magnified in lower panels. **b** Negative controls: PCa tissue sample stained only with secondary Ab; inset: lymph node negative for tumor cells stained with αV-integrin Ab. αV-integrin expression correlated with (**c**) Gleason score grading (**p = 0,005) and (**d**) lymph nodes metastasis (***p = 0,001)

In light of the results presented above, we investigated whether αV-integrin-positive LO-like vesicles could be also identified in human PCa tissue samples. As shown in Fig. 6, αV-integrin staining revealed vesicular structures in the size range of 1- to 10-μm in Gleason score 6 (GS6) and 8 (GS8) human PCa tissues as compared to no apparent detection in either normal prostate epithelium (NPE) or prostatic intraepithelial neoplasia (PIN) (Fig. 6a). This is more evident in the 60× lower panels and enlarged cut-outs. Several controls confirmed αV-integrin antibody specificity in both human and xenograft tumor samples (Fig. 6b; Additional file 7: Figure S6C).

Finally, to evaluate the potential prognostic impact of αV-integrin expression on PCa, we correlated αV-integrin expression on PCa tumor tissue with Gleason Score and lymph node status, leveraging a tissue microarray from a series of 103 radical prostatectomies, collected at IRCCS “Fondazione G. Pascale” of Naples. Patients were stratified according to Gleason score in two groups: 1) low Gleason score (≤ 6) (*n* = 32), 2) intermediate/high Gleason grade (≥ 7) (*n* = 71) (Additional file 8: Table S1). IHC staining highlighted αV-integrin expression both in the cytoplasm and on the plasma membrane of cancer cells, with variable expression in tumors and in healthy controls (*data not shown*). However, αV-integrin expression resulted statistically significant associated high Gleason grade being found in 66 tumors (92%), compared to low Gleason grade tumors (*p* = 0,005) (Fig. 6c). A statistically significant correlation between lymph node status and αV-integrin expression was also demonstrated (*p* < 0.001) in patients with lymph node metastases, which were 26% of the total (Fig. 6d).

## Discussion

EVs are important intercellular mediators of both cancer development and progression [9] and recent reports point to the possibility that different populations of EVs might activate distinct pathways. Therefore, a thorough characterization of each EV population [9] might be helpful to shed light on the mechanistic interaction between EVs and recipient cells, aiming to identify selective markers or therapeutic targets in different cancer types [24, 25]. A specific class of EVs, reported to be atypically large and cancer specific and known as LO [25], was reported for the first time in 2009 [10] and further characterized in PCa models [11–13, 26, 27]. LO shedding has been previously reported as promoted by both an amoeboid cell behavior, caused by silencing of the Diaphanous-Related Formin 3 (DIAPH3) [10], and activation of the EGFR-AKT pathway, via EGF stimulation, or upon overexpression of a membrane-targeted constitutively active form of AKT [9]. However, the distinct functional role of LO in cancer development and progression is far to be defined.

In our study by taking advantage of the unique isogenic model consisting of parental DU145 cell line and of DU145R80 aggressive derived subline, developed by our group as resistant to ZOL [14, 17], we described, for the first time to our knowledge, the occurrence of a high spontaneous blebbing/LO shedding in a PCa model. The spontaneous increased LO biogenesis we reported in DU15R80, was apparently independent of the mechanisms previously described and we hypothesized to be associated with the aggressive feature of this cell line and to the capacity to transfer these features to recipient cancer cells. In support of our hypothesis, we observed also in vivo the aggressive feature of DU145R80 cells, most likely correlated with the biological function of LO shed by this cell line. Indeed LOR80 were also able to promote tumor engraftment and development in DU145 recipient cells, that normally do not engraft in xenograft model without the support of matrigel [28].

The phenotype of DU145R80 we have described in a previous study, characterized by the reorganization of the F-actin cytoskeleton, and consistent with the identified differentially expressed proteins compared with the parental DU145 line, well correlated with increased LO shedding. Whether the resistance to ZOL, involving the mevalonate pathway, correlates with the increased LO shedding and the distinct features of LOR80, is under investigation. Intriguingly, RHOA, a small GTPase, whose activity is regulated by the mevalonate pathway, has been involved in the formation and shedding of plasma-membrane-derived EVs in cancer cells by activating a specific signaling [29].

We also showed that a selective packaging of proteins occurs within LOR80, directly correlated with the cell of origin and functionally conditioning their phenotype and that of other recipient cells. Notably, the most important finding of our study is the first evidence that αV-integrin overexpression at the surface of LOR80 is functionally involved in promoting adhesion and invasion, as well as the activation of FAK-AKT axis in recipient cells, shedding light on new mechanisms of PCa cancer development and progression. Integrins-dependent mechanisms have been associated to multiple steps of cancer progression and αV-integrin is considered a key-molecule in the invasion process in several cancers including PCa [6, 21, 30]. In this regard, we have previously correlated αV-integrin with the augmented aggressiveness of DU145R80 subline compared with parental DU145 cells [17]. Indeed, high αV-integrin tumor levels was recently correlated with PCa patient poor prognosis [30]. Moreover, αV-integrin has been recently associated to PCa self-renewal capacity and drug-resistance [30]. Interestingly, integrins were described among the most expressed receptors on the surface of exosomes and recently, a specific repertoire of integrins on tumor-derived exosomes, including αVβ5, were described as able to determine organ tropism for metastatic cells, by triggering in stroma target cells signaling pathway to prepare the pre-metastatic niche [6]. Notably, targeting these integrins, decreased exosome uptake as well as metastasis [6]. A previous report has identified αV-integrin among the proteins enriched in nanosized EVs as compared to LO produced by DU145 silenced for DIAPH3 [13], and αVβ6-integrin has been shown to be transferred by exosomes to heterologous cells [31]. However, as reported above, this latter cell model is distinct from DU145R80 cells that spontaneously showed blebbing and shed larger EVs than the parental counterpart. Anyhow, although all our experiments were performed using LO-enriched EVs preparation, a functional comparison of different type of DU145R80-derived EVs, including exosomes, warrants future investigations. Similarly, we cannot exclude that additional integrins might also play a role in our model and we are currently evaluating this hypothesis.

Among LOR80 protein cargo, we also found uPAR, another protein differentially expressed in DU145R80 cells compared to DU145 [17], with pivotal role in cancer adhesion, invasion and metastasis, related to its proteolytic activity as well as to the capacity to trigger changes in cell morphology, migration and signaling, by interacting with several molecular partners, including integrins [32–35]. Notably, uPAR has been related with PCa aggressiveness [17, 36] and its interaction with integrins has been previously described to control plasticity of PCa cell movement in both mesenchymal and amoeboid migration style [37], in line with the peculiar mesenchymal phenotype of DU145R80 we have previously described [14], as well as with blebbing, a typical amoeboid feature [38, 39]. In this regard, we demonstrated that both αV-integrin and uPAR co-localize with the LO-marker CK18 at the surface of blebs/LO-like structure arising from the plasma surface membrane of DU145R80. The finding that DU145 cell adhesion in the presence of both LO145 and LOR80 was reduced below the basal level when the integrin activity was fully inhibited may be attributable to a more complex interplay occurring between uPAR and αV-integrin that deserves further investigations.

A critical aspect to define is whether EVs are capable of docking on a cell and transferring their cargo inside the recipient cells or the docking event is sufficient to initiate signaling pathway that causes changes in recipient cells. Minciacchi et al. have recently demonstrated that AKT activity is critical for MYC-dependent reprogramming of stroma recipient cells upon the uptake of PCa-derived LO [12]. They also found active AKT in LO derived from PCa cells and from PCa patients plasma, however whether AKT activation in recipient cells was the results of LO cargo release, or the consequence of endogenous molecule activation upon “outside-in” signaling pathway in response to LO docking/uptake, remains an open question. In our study, although LO internalization by recipient cells cannot be excluded, we demonstrated for the first time the functional involvement of αV-integrin on LO surface in enhancing the adhesion and invasion of recipient cells via the activation of the AKT-dependent pathway. Indeed pre-incubation of LO with the αV-integrin blocking antibody reverted FAK and AKT activation, as well as pre-treatment of recipient cells with a small molecule AKT inhibitor was able to completely revert the pro-adhesive and pro-invasive effect exerted by LOR80. Both FAK and AKT are canonical pathway activated by integrins in “outside-in” signaling during both adhesion and invasion processes [3]. Intriguingly, in PCa cells it was also shown an “inside-out” signaling linking directly AKT activity to the modulation of αVβ3-integrin affinity for the ECM, with resulting alteration of the invasive and metastatic potential [40].

EVs containing matrix metalloproteinases (MMPs) and their secretion have been also shown to play a critical role in the interaction with recipient cells [41]. We provided evidences demonstrating that MMPs trafficking and activity can contribute to the pro-invasive effect promoted by LOR80 exposure of recipient cells, even independently of αV-integrin expression. LOR80 compared to LO145, contain high levels of MMP2, reflecting our previous observations demonstrating increased MMP2 transcription and secretion by DU145R80 compared to DU145 cells [14]. LOR80 also exhibit higher direct gelatinase activity, a characteristic previously reported in LO from different PCa cell lines [11]. However we also showed increased MMPs transcription and secreted activity by recipient cells upon exposure with LOR80, that at least in part, is reverted by the αV-integrin blocking antibody, corroborating the idea that the interaction of LO with DU145 recipient cells causes the activation of complex multiple pro-adhesive and pro-invasive pathways that are far to be completely defined. Indeed both MMP2 and MMP9 expression has been shown to be regulated via AKT dependent pathway [42–44]. Moreover, MMP2 as well αV-integrin and uPAR, all found among the protein cargo components overexpressed in LOR80, were identified as direct interactors of VN, a critical component of the ECM we have used in our functional experiments [20]. Intriguingly, interaction of MMP2 and αVβ3-integrin on the cell surface of cancer cells has been shown to induce the downstream PI3K/AKT signaling pathway [45]. Finally, we proposed that αV-integrin positive LO-like EVs could be used as a novel prognostic PCa biomarker. Indeed, in mice xenograft tumor tissues, we were able to identify LO-like structures efficiently detected for the first time by αV-integrin. More importantly, αV-integrin-positive LO-like structures were also detected on human PCa tissue samples, where αV-integrin-positive staining significantly correlated with high Gleason grade and lymph node status. This latter observation is in line with a recent report [30] and should be further validated on a large cohort of PCa patients.

## Conclusions

Overall, adding new insights in biological function of LO, we highlighted a novel autocrine/paracrine mechanism contributing to PCa development and progression that can be exploited for the selection of novel biomarkers and therapeutic targets. Our novel cytofluorimetric approach to detect and characterize intact/vital LO, positive for surface-expressed αV-integrin and/or other identified markers, could be further potentially tested to investigate liquid biopsies from PCa patients with the advantage of a less invasive intervention compared to tissue biopsy also allowing dynamic monitoring.

## Additional files

Additional file 1: Supplementary Material and Methods. (DOCX 23 kb)

Additional file 2: **Figure S1**. Serum free culturing conditions do not induce apoptosis in DU145R80. (A) Apoptosis, evaluated by Annexin-V binding on DU145R80 cells, cultured for 24 h in complete media (CTR), serum free media (SFM) or PBS. The values, expressed as fold changes of control, are the means ± S.D. from at least three independent experiments. (PDF 84 kb)

Additional file 3: **Figure S2**. Surface integrin profile in DU145 compared to DU145R80 cells. Integrins profile on both DU145 and DU145R80 cell surface. Results are representative of a single experiment performed in triplicate. SD are reported. At least three experiments yielded similar results. (PDF 109 kb)

Additional file 4: **Figure S3**. AKT activation upon LOR80 exposure is essential to LO-induced recipient cell adhesion. **(A)** Western blot analysis for the indicated proteins (30 μg/line) on DU145 cells treated/untreated with either LO145 or LOR80 for 15 min and 60 min. GAPDH served as loading control. **(B)** Western blot analysis for the indicated proteins (30 μg/line) on DU145 treated/untreated with 50 ng/mL EGF, following pretreatment with GDC-0068 for 6 h or 16 h. Ponceau served as loading control. **(C)** Adhesion was measured at 1 h in LNCaP cells, untreated/pre-treated for 16 h with the AKT inhibitor GDC-0068 and then treated with either LO145 or LOR80 or PBS as vehicle/control (not shown in the histogram, considered as 100%). Efficacy of treatment was analyzed as % of adherent cells. (PDF 104 kb)

Additional file 5: **Figure S4**. Invasion of diverse prostate recipient cells upon LO exposure (**A**) Invasion was performed on DU145 cells treated, as indicated in figure, with LO145 untreated or pre-exposed to blocking anti- αV-integrin antibody or to CTR Ab (anti-pAKT antibody ineffective on non-permeabilized intact cells). Results, shown as % of invading cells compared to the vehicle (reported as bar at 100%), are representative of a single experiment performed in triplicates. Several experiments yielded similar results. (**B**) Invasion was performed at 16 h on both epithelial normal prostate cells (EPN) and PCa LNCaP cells, untreated/treated with LO from both DU145 and DU145R80 cells. Results, reported as fold change of treated cells compared to the vehicle, are representative of a single experiment performed in triplicate and several experiments yielded similar results. (PDF 42 kb)

Additional file 6: **Figure S5**. LO induce metalloproteinases activity. (**A**) LO145 or LOR80 treatment effect on matrix metalloproteinase 9 (MMP9) mRNA expression in DU145 recipient cells. Results are expressed as fold changes of LO-treated DU145 compared to PBS treated DU145 (indicated as =1). The values are the means ± S.D. from at least three independent experiments. (**B**) Gel zymography assay performed on supernatants from DU145 treated with PBS as vehicle, LO145, LOR80 and 0.5% EtOh-treated DU145 as positive control. (**C**) Levels of MMPs, as indicated in figures, in DU145 supernatants after treatment with: LO145 (20 μg/mL), LO145 + mAb anti αV-integrin 1:500, LOR80 (20 μg/mL), LOR80 + mAb anti αV-integrin 1:500. MMPs levels were determined using a Bio-Plex array reader. Data are reported as % of decreased levels of single MMPs upon LO pre-exposure with the αV-integrin blocking antibody vs the expression evaluated in LO-treated DU145 (indicated as =1). The values are the means ± S.D. from a single experiment performed in triplicates. (**D**) Fluorescent gelatin matrix exposed to LOR80, compared to LO145. PBS (CTR) was used as negative control. Proteolitic spots are visualized. Proteases inhibitors cocktail (P.I.) to impair gelatine degradation by LOR80. (**E**) Size distribution of degradation spots, induced by LO and counted by Image J. (**F**) Zones of proteolytic clearance in the fluorescent gelatin, expressed as percentage of degradation spots. (PDF 159 kb)

Additional file 7: **Figure S6**. Kinetic of xenograft tumor growth in nude mice and tumor sample analysis. (A) Hematoxylin and eosin and (B) tumor volume curves of xenograft indicated tumors in nude mice. Negative αV-integrin staining on mice (C) normal lymph node (D) lung ((E) xenograft tumor sample (DU145R80 group) incubated only with secondary antibody. (PDF 186 kb)

Additional file 8: **Table S1**. Clinicopathological features of PCa patients shown in Fig. 6 (B-C panels). (DOCX 56 kb)

## Data Availability

All data generated or analysed during the present study are included in this published article.

## Abbreviations

CSC: cancer stem cells
DIAPH3: Diaphanous-Related Formin 3
eEF1γ: Eukaryotic elongation factor 1 gamma
EMT: epithelial to mesenchymal transition
EPN: normal epithelial prostate
EVs: extracellular vesicles
FAK: focal adhesion kinase
GS: Gleason score
IHC: immunohistochemistry
LO: Large Oncosomes
LO145: Large Oncosomes derived from DU145 cells
LOR80: Large Oncosomes derived from DU145R80 cells
MMP2: Matrix metalloproteinase 2
MMP9: matrix metalloproteinase 9
NPE: Normal prostate epithelium
PCa: Prostate cancer
PE: phycoerythrin
PIN: Prostatic intraepithelial neoplasia
TMA: Tissue microarray
TRPS: Tunable resistive pulse sensing
uPAR: Urokinase-type plasminogen activator receptor
VN: Vitronectin
αV-integrin: integrin alpha V

## Footnotes

1 Budillon et al., unpublished observations
