# Supplementary material for: Large oncosomes overexpressing integrin alpha-V promote prostate cancer adhesion and invasion via AKT activation
Source: J Exp Clin Cancer Res. 2019 Jul 18;38:317. doi: 10.1186/s13046-019-1317-6 (PMC6639931; doi:10.1186/s13046-019-1317-6)
Supplement: Supplementary file 1 — Supplementary Material and Methods. (DOCX 23 kb) [file 13046_2019_1317_MOESM1_ESM.docx]

**Supplementary Material and Methods.**

**Immunoblotting**

In details, for WB on cell lysates: pAKT (#9270), AKT (#9272), FAK (#3285) were all purchased by Cell Signaling and used at a concentration of 1:1000. y Tub (sc-17787, 1:1000) from Santa Cruz, while pFAK (ab81298, 1:1000) from Abcam. For WB on both LO and cells lysates: Cav-1 (cat. Orb10244, 1:500) was purchased from Biorbyt. CK8-18 (cat. #4546, 1:1000), GAPDH (cat. #5174, 1:1000), Lamin AC (cat #2032, 1:1000) were all purchased from Cell Signaling. MMP2 (MA1-16641, 1:500) was from Thermo Fisher; anti-uPAR monoclonal antibody recognizing uPAR D3 domain (1 μg/mL) [18]; eEF1γ from Novus Biologicals (NBP-91862, 1:1000); both αV-integrin (EPR16800, 1:1000) and TSG101 (ab30871, 1:1000) from Abcam. Secondary antibodies were purchased as follows: polyclonal swine anti-rabbit immunoglobulins/horseradish peroxidase (HRP)–linked IgG secondary antibody conjugate and polyclonal rabbit anti-goat immunoglobulins/HRP conjugate were from Dako-Cytomation (#P0217, 1:2000), and rabbit polyclonal anti-mouse IgG H&L HRP conjugate (#ab6728, 1:2000) was from Abcam.

**Real-time PCR**

After isolation of total RNA by Trizol® reagent and cDNA synthesis, qRT-PCR analyses were performed to quantify mRNA levels using the appropriate primers and the Applied Biosystems 7900 Real-Time PCR system. β-actin was used as housekeeping control genes.

**Gelatin FITC-assay**

The gelatin degradation assay was performed by seeding isolated particles (1 to 5 µg) on FITC-labeled gelatin-coated coverslips for 1.5h, at 37°C (11).

**Integrins Profile**

To profile integrins we took advantage of a colorimetric kit (α/β Integrin-mediated Cell Adhesion Array Combo Kit, Merck Millipore) following manufacturer's instructions. Briefly, following strips rehydration with 200 µL PBS/well, 100000 of each cell line (DU145 and DU145R80) were plated in serum free RPMI. After the indicated times serum free RPMI was removed, cells were washed and stained with a cell stain solution from the kit. Cell stain solution was removed and after several washes, stained cells were allowed to dry and then red at 540-570 nm.

**Gel Zimography**

Before starting the assay, we made the following solution: Washing Buffer (2,5% Triton X-100 in dH2O); Gel Development Buffer (50 mM Tris pH 7,4; 10 mM CaCl2; 0.02% NaN3); Gel Destaining Solution I (125 mL Ethanol, 50 mL acetic acid, 325 mL dH2O); Gel Destaining Solution II: 25 mL Ethanol, 37.5 mL acetic acid, 437.5 mL dH2O). 10000 cells of either DU145 and DU145R80 were plated in a 96 well plate. At 70–80% cell confluency, RPMI was removed and cells washed twice with serum free media. DU145 were treated with LO145 (20 µg/mL), LOR80 (20 µg/mL), PBS as LO-vehicle and Ethanol as positive control. DU145R80 were just added with PBS. After 8h treatment, surnatants from cells were removed, added with 5X non-reducing sample buffer and run in a 10% Ready Gel® Zymogram (#161-1167 Biorad). The gel was then washed 3 times with Washing Buffer, incubated for 42h in Development Buffer at 37° C and finally incubated with Blu di Comassie 1h a RT. Degradation areas were acquired by ImageQuant LAS 500 (GE Healthcare) after the gel was washed several hours with Destaining solution 1 and 2.

**MMPs profile**

Levels of MMPs (MMP-1, MMP-2, MMP-3, MMP-7, MMP-8, MMP-9, MMP-12, MMP-13, MMP-10) were evaluated at the same time with the Bio-Plex assay. The multiplex biometric ELISA-based immunoassay, containing dyed microspheres conjugated with a monoclonal antibody highly specific for a target protein, was used, according to the manufacturer’s instructions (Bio-Plex Bio-Rad), to evaluate the levels of different metalloproteases, in DU145 supernatants after treatment with: LO 145 (20 µg/mL), LO145 + mab antiav 1:500, LOR80 (20 µg/mL), LOR80 + mab antiav 1:500. Active protein levels were determined using a Bio-Plex array reader (Luminex, Austin, TX, USA) that quantifies multiplex immunoassays in a 96-well format with very small fluid volumes. The analyte level was calculated using a standard curve, with software provided by the manufacturer (Bio-Plex Manager Software).

**Immunohistochemistry Controls**

Negative controls of αV integrin signal in DU145 xenograft tumors as follows: a DU145R80 xenograft tumor sample was incubated with Novocastra Biotinylated Secondary Antibody (RE7103) alone; mice tissues that do not overexpress αV-integrin (normal lymph node and normal lung) were incubated with both αV integrin (EPR16800 Abcam Rabbit monoclonal IgG 1:500) and secondary antibody.

Negative controls of αV integrin signal in human PCa tissues as follows: a human PCa tumor was incubated with the secondary antibody Novocastra Biotinylated Secondary Antibody (RE7103) alone; human lymph node negative for tumor cells was incubated with αV-integrin (EPR16800 Abcam Rabbit monoclonal IgG 1:500) and secondary antibody.
