# Supplementary figures and images for: Large oncosomes overexpressing integrin alpha-V promote prostate cancer adhesion and invasion via AKT activation
Source: J Exp Clin Cancer Res. 2019 Jul 18;38:317. doi: 10.1186/s13046-019-1317-6 (PMC6639931; doi:10.1186/s13046-019-1317-6)

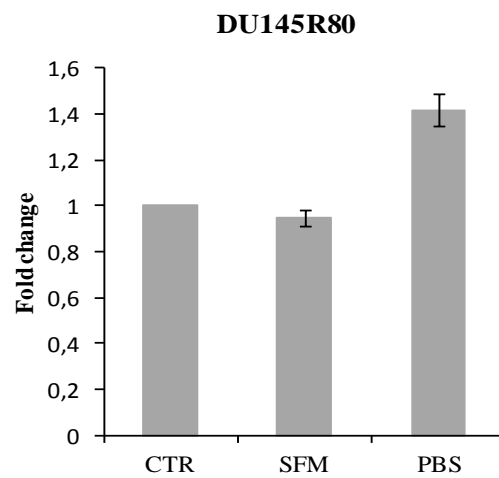

**Supplementary Figure S1. Serum free culturing conditions do not induce apoptosis in DU145R80.**

Supplement: Supplementary file 2 — Figure S1. Serum free culturing conditions do not induce apoptosis in DU145R80. (A) Apoptosis, evaluated by Annexin-V binding on DU145R80 cells, cultured for 24 h in complete media (CTR), serum free media (SFM) or PBS. The values, expressed as fold changes of control, are the means ± S.D. from at least three independent experiments. (PDF 84 kb) [file 13046_2019_1317_MOESM2_ESM.pdf]

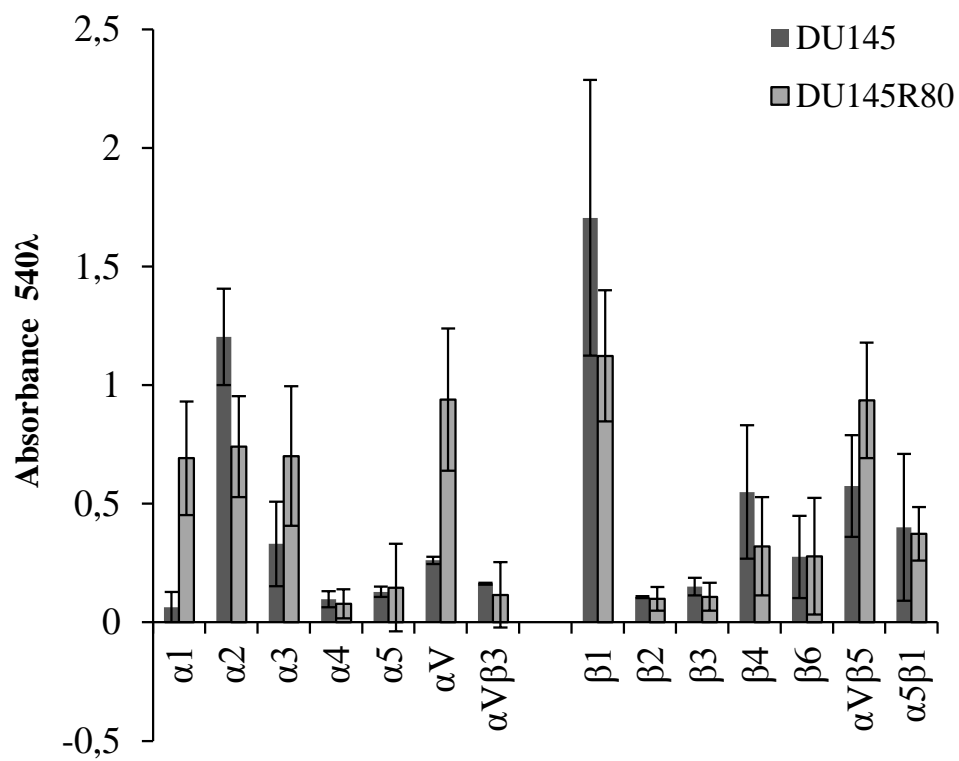

**Supplementary Figure S2. Surface integrin profile in DU145 compared to DU145R80 cells.**

Supplement: Supplementary file 3 — Figure S2. Surface integrin profile in DU145 compared to DU145R80 cells. Integrins profile on both DU145 and DU145R80 cell surface. Results are representative of a single experiment performed in triplicate. SD are reported. At least three experiments yielded similar results. (PDF 109 kb) [file 13046_2019_1317_MOESM3_ESM.pdf]

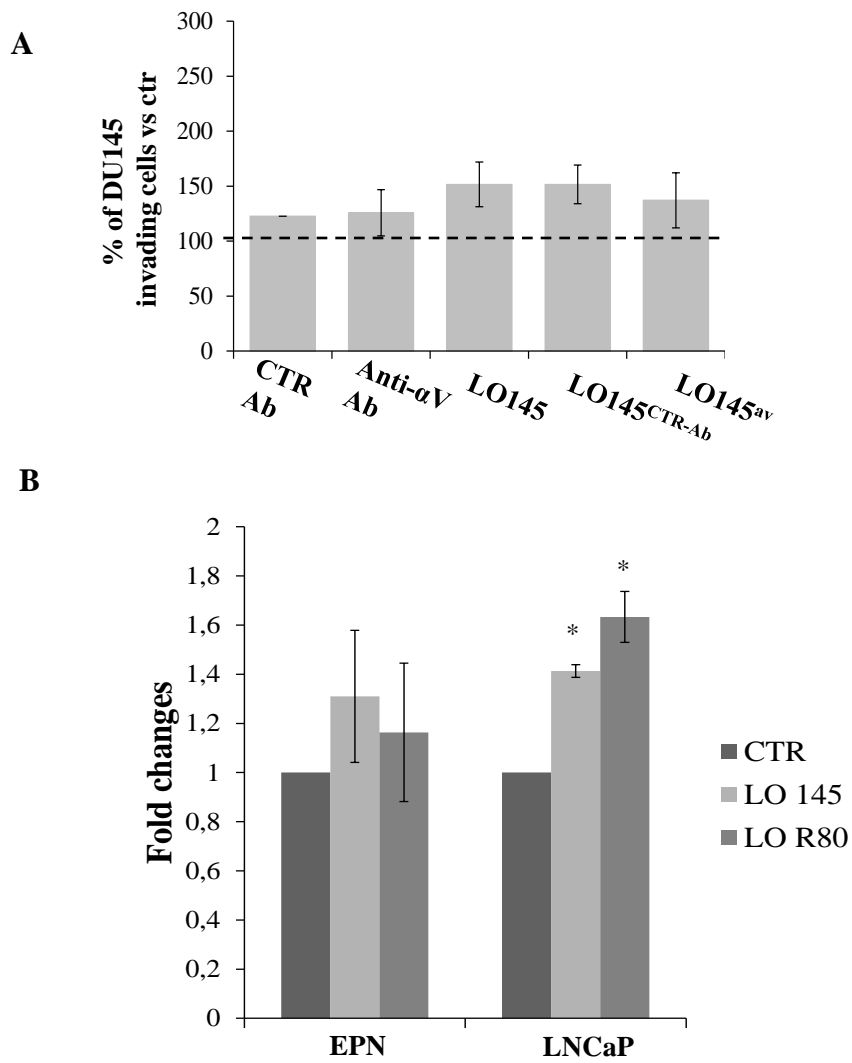

**Supplementary Figure S4. Invasion of diverse prostate recipient cells upon LO exposure**

Supplement: Supplementary file 5 — Figure S4. Invasion of diverse prostate recipient cells upon LO exposure (A) Invasion was performed on DU145 cells treated, as indicated in figure, with LO145 untreated or pre-exposed to blocking anti- αV-integrin antibody or to CTR Ab (anti-pAKT antibody ineffective on non-permeabilized intact cells). Results, shown as % of invading cells compared to the vehicle (reported as bar at 100%), are representative of a single experiment performed in triplicates. Several experiments yielded similar results. (B) Invasion was performed at 16 h on both epithelial normal prostate cells (EPN) and PCa LNCaP cells, untreated/treated with LO from both DU145 and DU145R80 cells. Results, reported as fold change of treated cells compared to the vehicle, are representative of a single experiment performed in triplicate and several experiments yielded similar results. (PDF 42 kb) [file 13046_2019_1317_MOESM5_ESM.pdf]

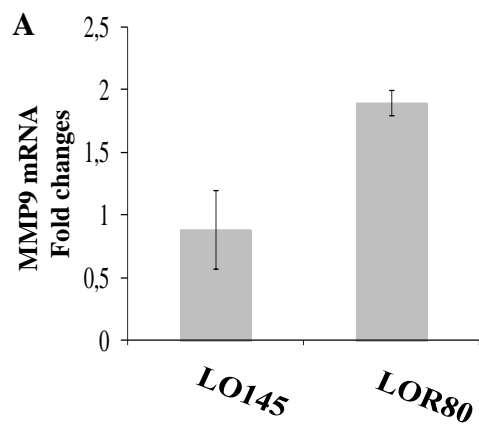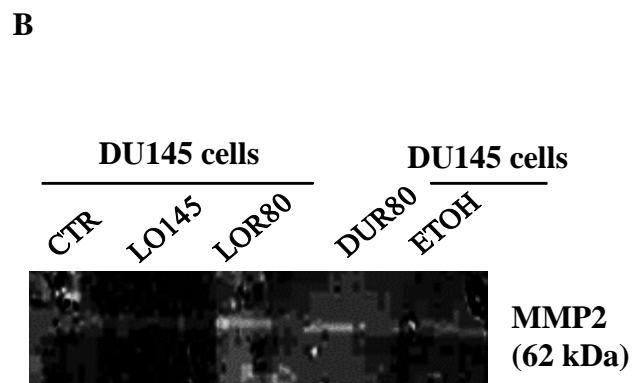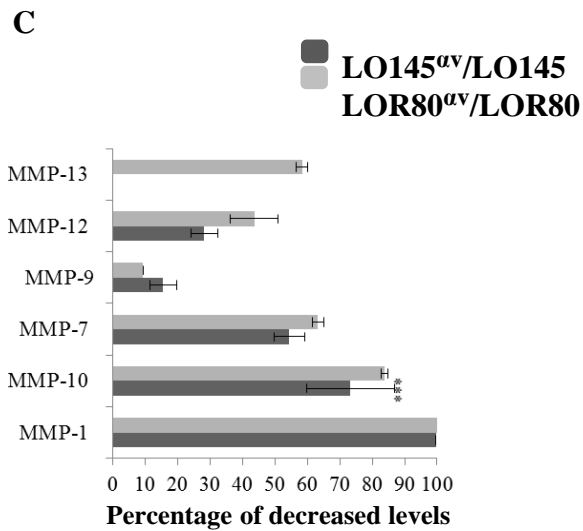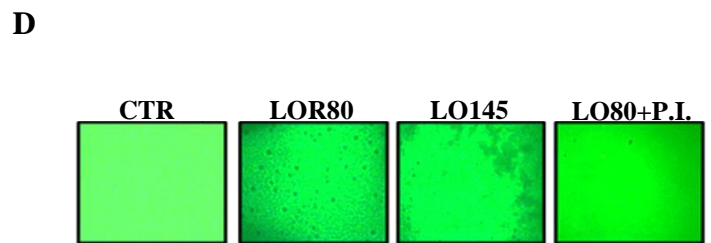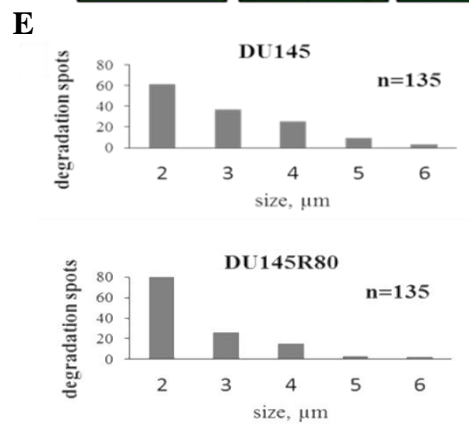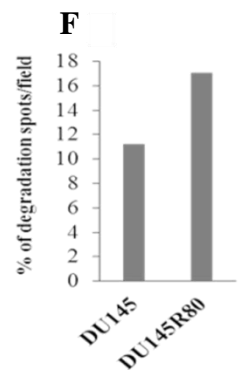

Supplementary Figure S5. LO induce metalloproteinases activity.

Supplement: Supplementary file 6 — Figure S5. LO induce metalloproteinases activity. (A) LO145 or LOR80 treatment effect on matrix metalloproteinase 9 (MMP9) mRNA expression in DU145 recipient cells. Results are expressed as fold changes of LO-treated DU145 compared to PBS treated DU145 (indicated as =1). The values are the means ± S.D. from at least three independent experiments. (B) Gel zymography assay performed on supernatants from DU145 treated with PBS as vehicle, LO145, LOR80 and 0.5% EtOh-treated DU145 as positive control. (C) Levels of MMPs, as indicated in figures, in DU145 supernatants after treatment with: LO145 (20 μg/mL), LO145 + mAb anti αV-integrin 1:500, LOR80 (20 μg/mL), LOR80 + mAb anti αV-integrin 1:500. MMPs levels were determined using a Bio-Plex array reader. Data are reported as % of decreased levels of single MMPs upon LO pre-exposure with the αV-integrin blocking antibody vs the expression evaluated in LO-treated DU145 (indicated as =1). The values are the means ± S.D. from a single experiment performed in triplicates. (D) Fluorescent gelatin matrix exposed to LOR80, compared to LO145. PBS (CTR) was used as negative control. Proteolitic spots are visualized. Proteases inhibitors cocktail (P.I.) to impair gelatine degradation by LOR80. (E) Size distribution of degradation spots, induced by LO and counted by Image J. (F) Zones of proteolytic clearance in the fluorescent gelatin, expressed as percentage of degradation spots. (PDF 159 kb) [file 13046_2019_1317_MOESM6_ESM.pdf]
