# Supplementary material for: Large oncosomes overexpressing integrin alpha-V promote prostate cancer adhesion and invasion via AKT activation
Source: J Exp Clin Cancer Res. 2019 Jul 18;38:317. doi: 10.1186/s13046-019-1317-6 (PMC6639931; doi:10.1186/s13046-019-1317-6)
Supplement: Supplementary file 4 — Figure S3. AKT activation upon LOR80 exposure is essential to LO-induced recipient cell adhesion. (A) Western blot analysis for the indicated proteins (30 μg/line) on DU145 cells treated/untreated with either LO145 or LOR80 for 15 min and 60 min. GAPDH served as loading control. (B) Western blot analysis for the indicated proteins (30 μg/line) on DU145 treated/untreated with 50 ng/mL EGF, following pretreatment with GDC-0068 for 6 h or 16 h. Ponceau served as loading control. (C) Adhesion was measured at 1 h in LNCaP cells, untreated/pre-treated for 16 h with the AKT inhibitor GDC-0068 and then treated with either LO145 or LOR80 or PBS as vehicle/control (not shown in the histogram, considered as 100%). Efficacy of treatment was analyzed as % of adherent cells. (PDF 104 kb) [file 13046_2019_1317_MOESM4_ESM.pdf]

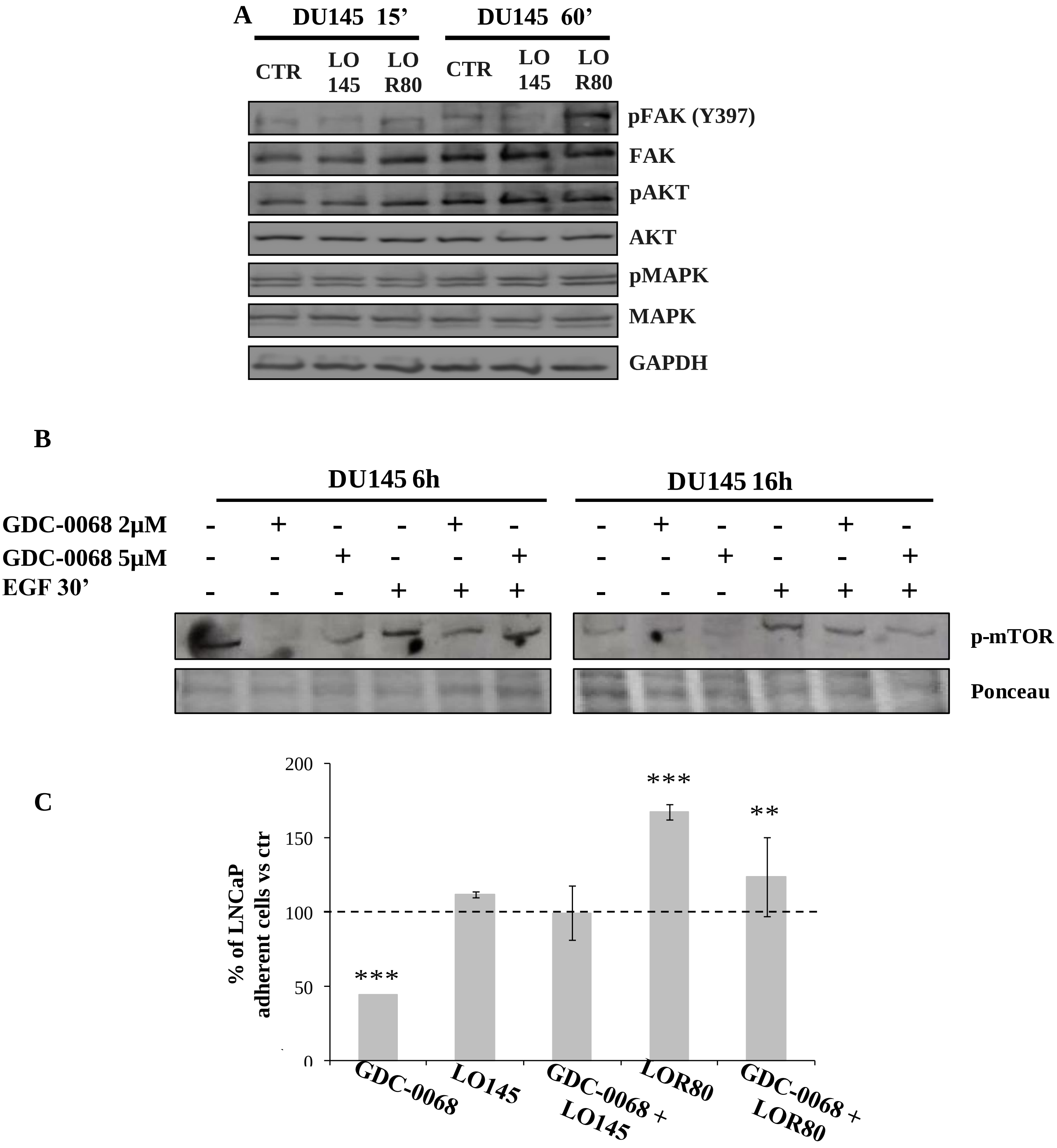

Supplementary Figure S3. AKT activation upon LOR80 exposure is essential to LO-induced recipient cell adhesion.
