# Supplementary material for: Large oncosomes overexpressing integrin alpha-V promote prostate cancer adhesion and invasion via AKT activation
Source: J Exp Clin Cancer Res. 2019 Jul 18;38:317. doi: 10.1186/s13046-019-1317-6 (PMC6639931; doi:10.1186/s13046-019-1317-6)
Supplement: Supplementary file 7 — Figure S6. Kinetic of xenograft tumor growth in nude mice and tumor sample analysis. (A) Hematoxylin and eosin and (B) tumor volume curves of xenograft indicated tumors in nude mice. Negative αV-integrin staining on mice (C) normal lymph node (D) lung ((E) xenograft tumor sample (DU145R80 group) incubated only with secondary antibody. (PDF 186 kb) [file 13046_2019_1317_MOESM7_ESM.pdf]

A

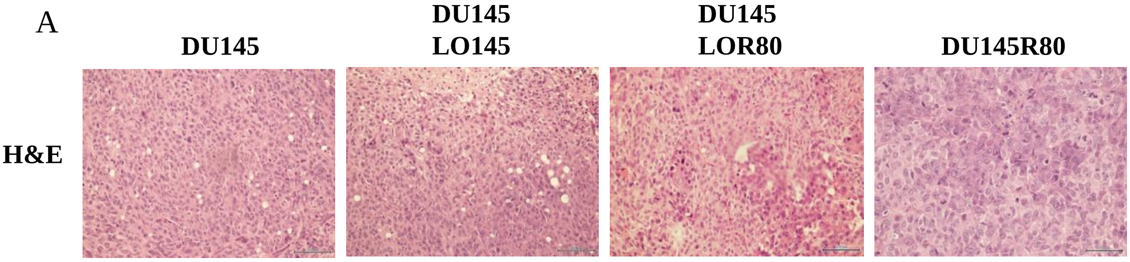

B

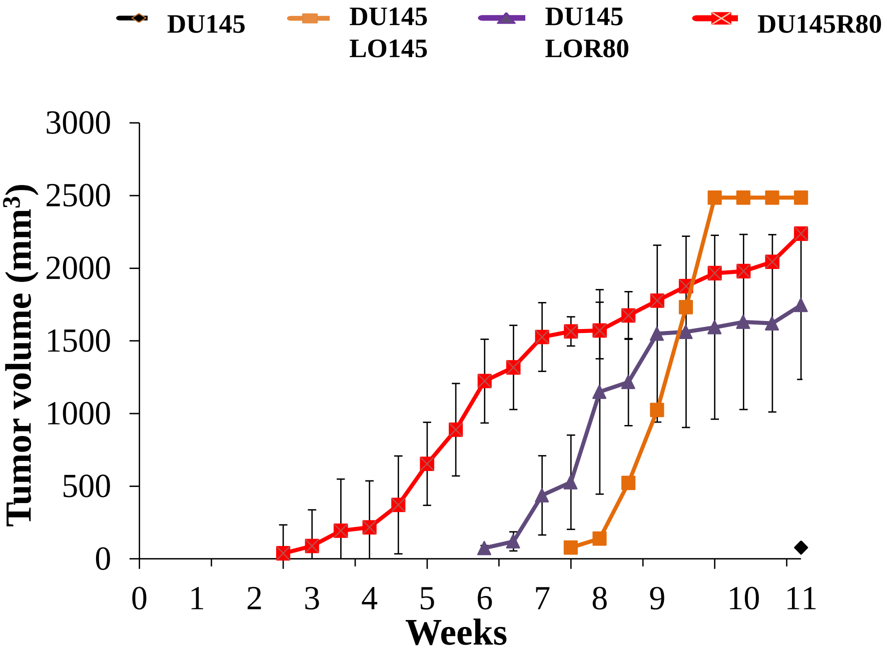

C D E

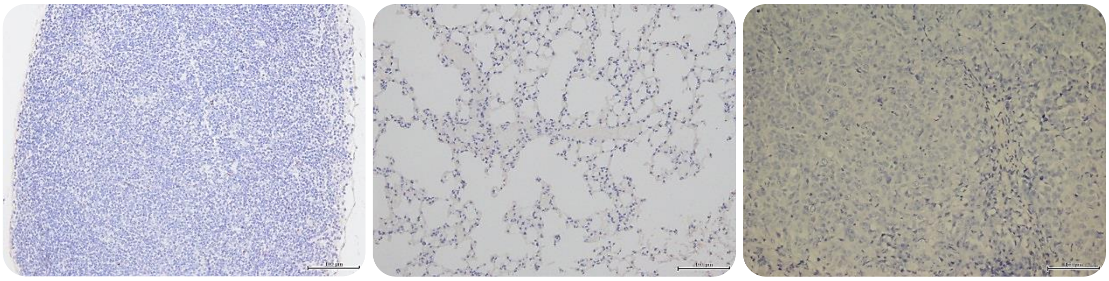

Supplementary Figure S6. A. Kinetic of xenograft tumor growth in nude mice and tumor sample analysis.
