# Supplementary material for: Large oncosomes overexpressing integrin alpha-V promote prostate cancer adhesion and invasion via AKT activation
Source: J Exp Clin Cancer Res. 2019 Jul 18;38:317. doi: 10.1186/s13046-019-1317-6 (PMC6639931; doi:10.1186/s13046-019-1317-6)
Supplement: Supplementary file 8 — Table S1. Clinicopathological features of PCa patients shown in Fig. 6 (B-C panels). (DOCX 56 kb) [file 13046_2019_1317_MOESM8_ESM.docx]

| **Characteristics** | **Number of patients** | **Percentage of patients** |
| --- | --- | --- |
| **Age at Diagnosis**  **> 65**  **≤ 65** | **57**  **46** | **55%**  **45%** |
| **Gleason Score**  **≤6**  **≥7** | **32**  **71** | **31%**  **69%** |
| **Tumor Stage**  **I**  **II**  **III**  **IV**  **NA** | **1**  **49**  **47**  **2**  **4** | **1%**  **48%**  **45%**  **2%**  **4%** |
| **Lymph node status+**  **Negative**  **Positive**  **NA*** | **63**  **27**  **13** | **61%**  **26%**  **13%** |
| **TOT** | **103** | **100%** |

**Supplementary Table 1.**

*NA= not available;

+Lymph node status: Negative= not metastatic, Positive= metastatic (N>0).
